# College and Hospital Notes

**Published:** 1910-10

**Authors:** 


### COLLEGE AND HOSPITAL NOTES

The Buffalo General Hospital was recently presented with a motor ambulance by Mrs. Charles W. Pardee. This is only one of Mrs. Pardee's many acts of sumptuous generosity toward the hospital. Mr. Pardee is president of the board of trustees, and to him also the hospital is indebted for many substantial gifts.

---

The University of Buffalo is to have a new laboratory, an order permitting it to mortgage its property on High street to the Erie County Savings Bank for $18,000 having been granted by Justice Woodward. The petition for leave to mortgage recites that the money is needed for a laboratory to be added to the medical college, which is growing so rapidly that more room is needed. The addition is to cost $20,000 and the balance will be supplied from funds on hand. The new mortgage will be a second lien, a balance of $18,900 remaining unpaid on a mortgage for $100,000 given by the university to the Erie County Savings Bank in 1892.

